# Cancer stem cells and macrophages: molecular connections and future perspectives against cancer

**DOI:** 10.18632/oncotarget.27870

**Published:** 2021-02-02

**Authors:** Beatrice Aramini, Valentina Masciale, Giulia Grisendi, Federico Banchelli, Roberto D’Amico, Antonino Maiorana, Uliano Morandi, Massimo Dominici, Khawaja Husnain Haider

**Affiliations:** ^1^Division of Thoracic Surgery, Department of Medical and Surgical Sciences, University of Modena and Reggio Emilia, Modena, Italy; ^2^Division of Oncology, Department of Medical and Surgical Sciences, University of Modena and Reggio Emilia, Modena, Italy; ^3^Center of Statistic, Department of Medical and Surgical Sciences, University of Modena and Reggio Emilia, Modena, Italy; ^4^Institute of Pathology, Department of Medical and Surgical Sciences, University of Modena and Reggio Emilia, Modena, Italy; ^5^Sulaiman AlRajhi Medical School, AlQaseem, Kingdom of Saudi Arabia

**Keywords:** cancer stem cells, macrophages, future perspectives, cancer, target treatments

## Abstract

Cancer stem cells (CSCs) have been considered the key drivers of cancer initiation and progression due to their unlimited self-renewal capacity and their ability to induce tumor formation. Macrophages, particularly tumor-associated macrophages (TAMs), establish a tumor microenvironment to protect and induce CSCs development and dissemination. Many studies in the past decade have been performed to understand the molecular mediators of CSCs and TAMs, and several studies have elucidated the complex crosstalk that occurs between these two cell types. The aim of this review is to define the complex crosstalk between these two cell types and to highlight potential future anti-cancer strategies.

## INTRODUCTION

Cancer stem cells (CSCs) constitute a cancer cell subpopulation similar to the other stem cell types in terms of self-renewal and multilineage differentiation potential but drive tumor development besides heterogeneity and dissemination of cancer cells [1–9]. CSCs have been extensively studied, with some of these studies focusing on their identification and their origin from differentiated cancer cells due to microenvironment’s influence, which contributes to their heterogeneous phenotypes [6, 10].

Targeting CSCs for therapeutic purposes is a goal of the scientific community. Currently, cancer treatments target the bulk population of the tumor cells without identifying and targeting CSCs [11–14]. This inability of the contemporary therapeutic protocols is significant in treatment resistance and metastasis of cancer cells [15–19]. The significant problem in this regard is the lack of identification marker/s specific for CSCs. Bonnet and Dick were the first to report the existence of CSCs in the tumor in acute myeloid leukaemia (AML) samples [20]. They found that CD34+ CD38- subpopulation cells in leukemia were similar to normal hematopoietic stem cells (HSCs) and could initiate AML in immuno-deficient mice [20]. Since the publication of this report, CSCs have been extensively studied for specific markers but with little success [21, 22]. Nevertheless, CD34+CD38- expression differs between normal and CSCs and may be useful for the identification of CSCs [23, 24]. Receptors expressed in several cell types bind other molecules and be specific for normal, epithelial cancer cells, and CSCs [25, 26]. In the recent decades, scientists have often discussed the identification of CSCs and the use of a specific superficial target that can identify CSCs is the most commonly used approach [27–35].

The main characteristic of these antigens is the capacity to target an endogenous stem cell. However, a standard marker specific for CSCs has not yet been found, although several gene markers have been recently described for CSCs in different tumors, including brain, breast, blood, and lung [36–41]. One of the major problems in finding such a marker is that several markers are able to detect not only CSCs, but also non-tumor cells, which represents an obstacle in developing new therapies targeting CSCs [42–44].

Indeed, there are currently no markers able to distinguish between stem cells and CSCs. Thus far, the best markers identified are those of onco-fetal stem cells, which are absent in adult organs and present in cancer cells [45–48]. Most of the markers identifying stem cells are proteins or glycoproteins, i.e., CD15, an embryogenic target in glioblastoma [49–51]. These identification markers may be subjected to genetic modifications and identify specific CSCs subpopulations with different molecular characteristics. For example, the upregulated ATP-binding cassette (ABC) transporter G subfamily (ABCG), absence or presence of mutated NOTCH1, or different isotypes as observed for CD44v [33, 52–56].

Another essential characteristic of these markers is the variability in their localization as they may be on the cell membrane like CD133 or in the cytoplasm/nucleus like aldehyde dehydrogenase 1 (ALDH1), which is located in the cytoplasm and has been observed in several solid tumors as well as leukemia [57–61]. The localization and the characteristics of these markers reveal that CSCs may have different epigenetic and genetic alterations [62, 63]. This may be one of the most important reasons why scientists are still debating about possible CSCs markers in different solid tumors [45, 64]. Despite that, these aspects have been investigated in cell lines and experimental animals’ models for decades. Sullivan et al. (2010) described the role of ALDH as a possible marker for lung cancer stem cell, as ALDH+ cells in cancer cell lines, as well as those extracted from lung cancer tissue, for the properties that it showed as forming spheres in culture tumor cells lines as well as in cells extracted from lung cancer tissue [65]. However, currently there is no specific or standard marker in lung cancer cells that can define this subpopulation of cells, probably due to the complex localization of these markers and their epigenetic regulation.

Different markers for CSCs have been studied in the last recent decades, and scientists tried have attempted to improve the identification of this better this subpopulation by using a double or triple marker [15, 32, 33, 65–70]. Masciale et al. reported a novel subpopulation of cells expressing CD44+/epithelial cell adhesion molecule (EpCAM^+^) that was positively correlated with ALDH+ cells [33]. They compared two similar CSC populations. These significant data may form the basis to develop new targeted treatments to eliminate CSCs, which are considered to be one of the leading causes of tumour recurrence and progression [71–75].

A theory regarding the role of CSCs in cancer progression is based on the premise that tumor tissue is hierarchically organized into different types of cells wherein CSC subpopulation is at the top of this hierarchy [1, 28, 29], with the other levels consisting of more differentiated tumor cells or cells with a limited proliferative potential [30]. CSCs have many attributes, including quiescence, chemotherapeutic resistance, and slow cycling [74–76]. Another essential characteristic that places CSCs at the top of the tumor cell hierarchy is their unlimited proliferation potential, which allows them to repopulate the tumor even if bulk tumor cells have been removed [18, 77–87]. It is important to note that CSCs may represent a dynamic cellular state in which stem cell-like traits are acquired to mediate resistance and induce tumor dissemination [77, 86, 87]. The complexity of cell composition, which is the base of *cancer heterogeneity*, has been discussed for a long time due to the different mechanisms that are the cause of the cancerization process variability [89]. Moreover, the discovery of the plasticity of CSCs and the possibility of switching from stem to non-stem cells led to a more complex picture of the origin of tumor heterogeneity [88]. Peter Nowell was the first to describe the “clonal evolution theory,” defining cancer as a complex process resulting from the development of a single out-of-control cell with multiple cell mutations that result in the progression of the tumor, which is kept viable through the selection of the most aggressive clones [89]. He also hypothesized that the dominant clone cells showed the most substantial tumorigenic properties [89]. An opposing theory is based on the concept that CSCs are a group of cells endowed with a high self-renewal capacity that can set different phenotypes of tumorigenic cells [18, 88]. The CSC theory was the most impressive in solid tumors in the absence of another approach to distinguish between a tumorigenic and non-tumorigenic cell due to similarity in their kinetic properties and because cancer is hierarchically organized [21]. One of the main obstacles to proving the CSC model is the difficulty in identification and isolation of these cells [7, 33, 91]. In fact, scientists first tried to define a CSC model using xenografts for the testing and identification of markers, and isolation of tumor-initiating cells [91, 92]. This model showed the existence of highly tumorigenic cells but did not clarify the superficial markers that might be useful to define this subpopulation compared with non-CSCs. One of the most important aspects that require in-depth study in the future is the capacity of these cells to survive chemotherapy. In addition to these aspects demonstrated in an animal models, these cells’ self-renewal capacity, which helps the tumor grow, disseminate, and relapse remains a topic of intense interest in the scientific community. Although the CSC model alone is not supported enough to explain functional heterogeneity in cancer, scientists have recently considered the role of the tumor microenvironment (TME) as a significant factor in CSCs’ plasticity, especially in the process of turning from non-CSCs to CSCs [90, 92]. This mechanism seems to rely on cell-to-cell interactions within the tumor niche [77].

**Figure 1:**
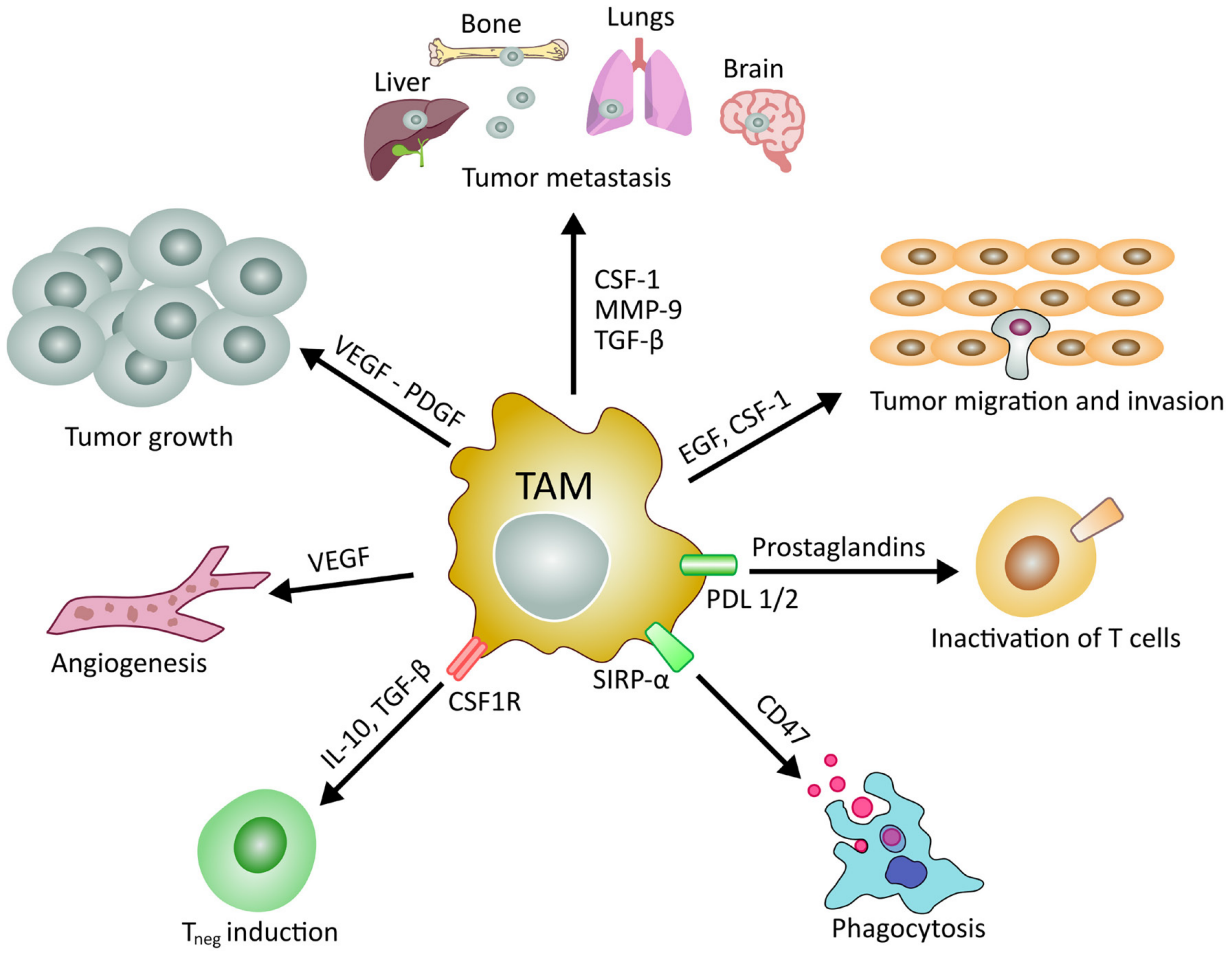
Main roles of tumor associated macrophages in cancer development and manteinance. TAMs and their released factors are involved in different processes controlling the evolution of cancer. Schematic representation of the role of TAMs in tumor growth, angiogenesis, invasion, metastasis and regulation of T lymphocytes.

Furthermore, these connections between CSCs and other cells is the primary source of protection for this subpopulation during induction of cell transformation, tumor growth, and resistance to common oncological treatments. In this context, the TME seems to play a crucial role in tumor progression and metastasis by building a synergistic relationship with CSCs [6, 10, 16, 76, 93] (Figure 1).

## CSCS ARE THE KEY DRIVERS OF TUMOR INITIATION AND PROGRESSION

The role of CSCs in carcinogenesis has not yet been well-defined [86, 87, 93, 94]. The process of tumor initiation involves the accumulation of mutations that facilitate the uncontrolled proliferation of tumor cells. Any mutation in these proliferation-promoting genes results in the failure of DNA-repair mechanisms [95, 96]. Additional mutations induce clonal selection, which selects more aggressive phenotypes. Unfortunately, these molecular alterations usually take place in the early stages of cancer that continue unabated in the absence of any treatment, as cancers are rarely identified in early stages [97]; thus, cancer grows and disseminate during this time unchecked [1, 98]. The most substantial support for metastasis comes from the TME, which provides favourable signals to support the metastatic cascade. The molecular interactions between cancer cells and the TME influence the tumor’s capacity to survive and evolve, developing more resistant and aggressive phenotypes [6, 10, 16, 76, 93]. This is the possible reason that cancer treatments are not able to reduce or stop tumor progression, especially in advanced stages. The tumour’s aggressiveness is primarily determined by the subpopulation of CSCs, as they are often resistant to therapies and can re-initiate tumor even if the bulk tumor cells are eliminated, resulting in tumor relapse [99].

The dualistic model explains this capacity to mediate tumour progression. By this model, CSCs may divide either symmetrically, giving rise to two identical CSCs or two differentiated cancer cells, or asymmetrically thus resulting in one CSC and one differentiated cancer cell. Only the complete extinction of the CSCs population would completely eradicate the tumor [100, 101]. Alternatively, if we consider the stochastic model in which each cell can be tumour-initiating, the path to eradicating the tumor is much more complicated [102]. Olmeda et al. put forth a tumor initiation model comprised of CSCs, differentiated cancer cells, and all the other cells within a tumour [103]. Each cell type in the tumour then creates an active link with the microenvironment. By this model, each cell subpopulation can actively interact with the microenvironment via diverse chemical pathways and physical interactions [103].

The CSC initiation process is stemness transcription factors dependent that drive the expression of genes not expressed in normal cells but is highly expressed in CSCs, especially during the initiation process [102–104]. Blanpain et al. demonstrated a vital role for the transcription factor sex-determining region Y-Box 2 (SOX2) in the melanoma initiation and progression [105, 106]. In this study, they noted that SOX2 was not present in normal skin but was clearly expressed at an early stage in tumor formation [106]. As these data, two now better understand these stemness genes and their potential targets during the control of tumor progression. In fact, in the case of melanoma, it has been thought that tumor initiation may be prevented by the deletion of the SOX2 gene and that the removal of SOX2-positive cells from established tumors may lead to regression. Potential treatments developed based on the study of stemness genes are very promising for controlling CSCs and, consequently, tumor resolution [106]. Future studies are required in many solid organs to identify the genes implicated in the mechanism regulating CSCs proliferation, tumor survival, and invasion [107].

## MACROPHAGES AND TAMS

Macrophages are large specialized phagocytic cells that exist in tissues or at infection sites. They arise from monocytes in the bone marrow and perform different functions and roles in the microenvironments of normal and tumor tissue [108, 109]. Macrophages differentiate into classically activated subtypes: CD68 expressing M1 mainly involved in pro-inflammatory activities, and CD163 expressing M2, that promote anti-inflammatory processes. In tumors, tumor-associated macrophages (TAM) comprise up to 50% of the tumor mass, with M2 phenotype being most abundant in the TME [110–112]. The primary signals provided by TAMs include interleukin 4 (IL-4) and transforming growth factor-beta (TGF-β). TAMs play a key role in tumor initiation, development, and cancer cell propagation [113] (Figure 1).

Recent studies have demonstrated that high numbers of TAMs correlate with a poor clinical prognosis in lung tumors and gastric cancer, among other cancer types [114–118]. Another important aspect is the protective role of TAMs for tumors undergoing chemotherapy, which may impact chemotherapeutic resistance and consequent tumor relapse [119]. There is a general reception that TAMs decrease the effectiveness of chemotherapy while the presence of CD68+ and CD163+ cells correlates with a poor prognosis in esophageal and pancreatic cancers [120, 121].

Yang et al. have suggested that in breast tumors, TAMs are responsible for chemotherapy drug resistance via the interleukin 10 (IL-10)/signal transducer and activator of transcription 3 (STAT3)/B-cell lymphoma 2 (Bcl-2) signaling pathway [122]. TAMs are also correlated with tamoxifen resistance, which is used in endocrine therapy in postmenopausal patients with breast cancer [122]. Moreover, TAMs contribute to unfavorable outcomes during radiotherapy due to their capacity to modulate cancer cells’ response to therapy [123, 124]. In particular, macrophages are responsible for the side effects of radiotherapy [123–125]. These findings explain that irradiation, DNA damage, cell death, and hypoxia stimulate tumors to produce vascular endothelial growth factor (VEGF), stromal cell-derived factor 1α (SDF-1α), and colony-stimulating factor 1 (CSF-1), which are involved in the recruitment of macrophages to the tumor [122–126] (Figure 1). The recruitment of TAMs leads to the production of proangiogenic cytokines, which stimulate blood vessel formation [121, 122]. Irradiated macrophages can also promote cancer cell migration and tumor angiogenesis (Figure 1). Notably, it has been demonstrated that therapies targeted against TAMs can improve radiotherapy’s efficacy [127–130]; specifically, the inhibition of TAM recruitment can prevent tumor regrowth [127].

## TUMOR ESCAPE AND TAMS

Immune system can recognize the presence of cancer cells and induce their rejection response. However, some specific phenotypes of cells manage to escape the vigilant immune surveillance to form tumors despite the presence of normal activated immune cells [131–133]. Immunoediting comprises all of the immune processes that lead to the control of tumor progression, including an important phase of immunosurveillance [134]. As a consequence of this tumor elimination phase, a specific subpopulation of CSCs is able to escape immune mechanisms to escape the immune response via, for example, the downregulation of antigen-presenting cells (APCs) [135, 136]. As the presentation of tumor antigens from human leukocyte antigen (HLA)-1 to T cells is essential for the recognition phase, the expression of HLA-1 on CSCs may decrease as reported by Di Tomaso et al. in a study on glioblastoma CSCs [137]. This downregulation of HLAs has also been observed in melanoma CSCs, in which abnormally high expression of HLA-II and low expression of the melanoma-associated antigens MART-1, ML-IAP, NY-ESO-1, and MAGE-A was observed [138, 139]. The antigen-processing machinery is defective in CSCs; thus, these highly specialized cells exhibit low immunogenicity [140]. Some CSCs, particularly in melanoma, modulate immune responses by expressing *ABCB5* gene, conferring chemoresistance [141–143]. These mechanisms mentioned above describe the modulation of immune response via the induction of T-cell anergy and downregulation of cancer-associated antigens to escape immune-mediated tumor clearance [141–143].

Another mechanism that can influence immune cells presence and function is associated with TAMs [121]. TAMs are the predominant immune cells within tumors; consequently, they significantly impact the tumor initiation process and can affect T lymphocytes, natural killer (NK) cells, dendritic cells, neutrophils, and myeloid-derived suppressor cells (MDSCs) [144]. Also, TAMs express chemokines, i.e., CCL5, CCL22, and CCL20 and cytokines i.e., IL-10 and TGF-β, which recruit and activate regulatory T cells (Tregs), thus contributing to immunosuppression in the TME [145], and participate in tumor cells’ escape and tumorigenesis. In particular, TAMs can suppress the antitumor effects of tumor-infiltrating T cells and NK cells [148] and inhibit T cell function by releasing specific enzymes, such as nitric oxide synthase and arginase I [146, 147].

Besides, TAMs inhibit the cytotoxic functions of T cells, natural killer T cells, and NK cells through the expression of the ligands for the immune checkpoint receptors programmed cell death-1 (PD-1) and cytotoxic T lymphocyte-associated protein 4 (CTLA-4) [121], which are highly expressed on the surface of CSCs in various cancer types, allowing CSCs to escape and avoid elimination by the immune cells [148].

Depending on their phenotype, macrophages have a dual role in cancer. M1 macrophages are involved in the earlier stages of neoplasia, while M2 macrophages are involved in tumor spreading [109]. DNA damage is the principal mechanism by which inflammation promotes tumorigenesis. TAM-generated free-radicals lead to DNA damage, causing alterations that predispose a cell to cancer. An example of this macrophage-mediated induction of tumorigenesis is Crohn’s disease, which significantly enhances the risk of colorectal cancer [149]. In the metastatic process, TAMs are responsible for local invasion and intravasation into the blood system, as well as homing to the pre-metastatic niche (by promoting EMT) [150, 151]. TAMs also foster both tumor growth and migration by producing a variety of chemokines, inflammatory agents, and growth factors. For example, CD68+HLA-DR+ TAMs in hepatocellular carcinoma (HCC) induce HCC cell migration *via* the nuclear factor kappa B (NF-κB)/ focal adhesion kinase pathway [109, 152]. One of the most exciting characteristics of TAMs is their capacity to promote angiogenesis and lymphangiogenesis that allow tumor growth and the spread of cells in the TME.

The formation of new blood vessels or lymphatic vessels provides support channels for neoplastic tissues. Massive angiogenesis contributes to poor prognosis in primary tumors. The principal contributors in angiogenesis are hypoxia, hyper-osmosis, and proangiogenic factors such as VEGF, TGF-β, cyclooxygenase 2, platelet-derived growth factor (PDGF), epidermal growth factor (EGF), angiopoietins, and chemokines [152]. TAMs can also synthesize proteins associated with vascular endothelial cells, such as Wnt7b besides VEGF, thereby promoting the angiogenetic switch [153]. One of the significant angiogenesis-inducing factors, pro-matrix metalloproteinase-9 (proMMP-9), is supplied by TAMs to the TME [156]. MMP-9 plays a fundamental role in tumor angiogenesis and metastasis by activating the angiogenic switch that mediates the development and maintenance of distinct neovascular networks [154, 155] (Figure 1).

Cross-talk between CSCs and TAMs involves the recruitment of TAMs through vascularization and the release of chemokines by TAMs to preserve the quiescence of CSCs and modification of their antigens to escape from recruitment by immune cells. This crosstalk influences all the aspects of tumorigenesis to metastasis. Consequently, immunotherapies to fight cancer, such as checkpoint inhibitors or T-lymphocyte transfer strategies are emerging novel therapeutic strategies in the oncology field.

## MECHANISMS LINKING CSCs AND TAMs

TAMs may constitute more than 50% of the tumor mass [109, 112, 156]. They promote tumor growth by inducing neoangiogenesis, supporting CSCs, and downregulating tumour-targeting immune cells’ number and function [125, 153–155]. Due to the significance of the tasks in which TAMs are involved, TAMs are increasingly becoming principal targets of novel therapeutic approaches, especially in the field of nanomedicine. It is now generally accepted that the M2 macrophages have an essential role in immunosuppression and trophic activity in response to Th2 cytokines [121, 156–159].

The roles, connections, and functions of the crosstalk between TAMs and CSCs have been studied in-depth during the recent past. [158–160]. The interactions may be direct or indirect, and the effects on CSCs include chemoresistance, preservation, and the capacity to differentiate [161, 162]. TAMs produce cytokines including milk fat globule epidermal growth factor 8 (MFG-E8); interleukin 6 (IL-6), which can activate STAT3; and the Hedgehog signaling pathway, which seems to be one of the causes of drug resistance [163, 164]. For example, in hepatocarcinoma, IL-6 promotes the expression of CD44+, inducing tumor development [165].

The role of IL-6 in the induction of TAM-mediated CSCs has been studied by the inhibition of IL-6 with the anti-IL6-R antibody tocilizumab, which was able to decrease the number of the tumor spheres and chemoresistant cells [166, 167]. IL-6 also plays a role in cancer by stimulating the conversion of non-stem cell into stem-like cells. In breast cancer, *in vitro* stimulation with IL-6 increases tumor mammospheres and CD44+/CD24+ breast CSCs. It is also known that IL-6 stimulates the conversion of non-stem cell into stem-like cells. In breast cancer, *in vitro* stimulation with IL-6 results in an increase in the number of tumor mammospheres and CD44+/CD24+ breast CSCs [168]. Substantial evidence demonstrates the important role of IL-6 in the niche microenvironment to guide the metastatic process in cancer [164, 165]. Moreover, IL-6 promotes stemness in osteosarcoma by upregulating the signalling pathway regulating EMT through phosphorylation of the STAT3. Further, IL-6 levels are associated with tumor growth and metastases [169–172]. The IL-6 release, together with CCL5 and IL-8, has been linked to the β-catenin/Wnt pathway, leading to the spread of CSCs [173, 174].

Another cytokine able to drive EMT in hepatoma cells is TGF-β1. This induction promotes the development of cancer stem-like characteristics, and it can be reduced or stopped by the depletion of TGF-β1 [175, 176]. A microarray from 96 patients diagnosed with pancreatic ductal adenocarcinoma showed CD44+CD133+ markers for CSCs and CD204+ for TAM, suggesting a possible connection between CSCs and TAMs in cancers, which also appears to be correlated with overall decreased disease-free survival [177]. Also, breast cancer CSCs appear to interact directly with TAMs through CD90/CD11b anchoring [178]. This interplay induces EphA4 receptor-mediated activation of both the nuclear factor- kappa B (NF-κB) and Src signaling pathways in CSCs. Cytokines and granulocyte-macrophage colony-stimulating factor (GM-CSF) are secreted by CSCs through a cascade of juxtracrine signals. However, the interactions between CSCs and TAMs may be facilitated by several components of the extracellular matrix (ECM). In particular, there are molecules like TGF-β1 responsible for EMT, which promotes the development of cells with cancer stem-like characteristics [167].

In leukemia, several cytokines, growth factors, and ECM proteins derived from mesenchymal stromal cells promote abnormal proliferation and dissemination of cancer cells [179]. Periostin (POSTN) is also involved in metastasis of different cancer types by promoting tumor niche formation, especially the CSC niche [179], and CCL2 was shown to increase the development of CSC in breast cancer by acting on cells in the TME [180, 181]. A recently identified pro-TME factor, Wnt-induced signaling protein 1 (WISP1) has been related with the promotion CSCs and TAMs’ survival of both in glioblastoma thus affecting the disease course [182, 183]. Moreover, in breast cancer, upregulation of hyaluronan synthase 2 (HAS2) induces the production of the hyaluronic component of the ECM, which is can promote TAMs-released platelet-derived growth factor-BB (PDGF-BB) [184]. In turn, PDGF-BB activates stromal cells, which induce CSCs’ self-renewal through the secretion of fibroblast growth factor 7 (FGF7) and FGF9. Inhibition of HAS2 by 4-methylumbelliferone blocks cancer development and reduces the incidence of recurrence [184]. This finding highlighted that inhibiting HAS2 can control the interactions between CSCs and TAMs. This could be considered very important in developing a new generation of therapeutic approaches targeting both of these cell populations [184].

TAMs also indirectly affect CSCs’ differentiation by NK cells by secreting IFN-γ [185, 186]. TAMs originate from monocytes upon their activation in the TME. The tumor actively recruits monocytes and favor the M1-to-M2 conversion, a local tumor-associated event that becomes more frequent with tumor development [112, 121, 156, 157, 187]. Efforts are underway to reprogram or inhibit the tumor-protective properties of TAMs, and develop potential strategies to increase the efficacy of conventional chemotherapy by combining it with macrophage-associated delivery of nano-drugs [121, 156, 157, 187]. The potential link between monocyte activation and macrophage conversion into TAMs, and the interactions between M2 macrophages and CSCs, are not well understood. It appears that CSCs should promote the conversion from M1 to M2, induce neo-vascularisation via VEGF release, and create CSCs niches through tissue repair pathways [188]. Molecular studies have demonstrated a crosstalk between TAMS and CSCs wherein TAMs release milk fat globule–EGF factor 8to activate the CSC-associated pathways STAT3 and Shh and amplifies the drug resistance and tumorigenicity of CSCs [189]. Indeed, the drug resistance of murine mammary CSCs was linked with the EGFR/STAT3/SOX2 signaling pathway by Yang et al., who reported paracrine activity established through a complex interplay between CSCs and TAMs [189–191].

In glioma, macrophages of the microglia and brain also produce high levels of TGF-β, thus rendering glioma stem-like cells (GSLCs) more invasive. This was accompanied by a substantial amount of MMP-9, a serine protease that contributed to the invasiveness of GSLCs [191–193]. However, there is insufficient data to identify all of the tumor-associated factors engaged in macrophages’ conversion to TAMs. The participation of M2 macrophages in tumor development is similar to their role in wound healing. The wound healing process has four programmed phases including hemostasis, inflammation, proliferation, and remodeling [194]. Wounds trigger mobilization of bone marrow MSCs and EPCs involved in neovascularization. These steps share similarities with tumorigenesis, in which CSCs initiate the formation of the primary tumor or metastatic nodes and, perhaps, play an essential role in the M1-to-M2 conversion. Therefore, tumorigenesis is often considered as a deviated natural healing process involving the participation of transformed stem cells (CSCs) and macrophages (TAMs) [109, 156]. CSCs release factors that attract macrophages and convert them into TAMs. CSF-1, a significant growth factor involved in this process, helps recruit macrophages to the tumor site, promoting tumor progression to malignancy. Inhibition of TAM recruitment by a CSF-1 may likely improve the ability of chemotherapeutic agents to reduce tumor progression and metastasis [109, 156, 195, 196]. Resting CSCs populate the hypoxic areas of tumor and get activated after chemotherapy-induced injury, when most peripheral cancer cells are eliminated. When macrophages are recruited to remove debris, they activate dormant CSCs. TAMs participate in reparative mechanisms after radiotherapy or antiangiogenic treatment [152–154]. Depending on various factors, they either enhance or antagonize the efficacy of radiotherapy or chemotherapy and immunotherapeutic agents such as tumor-targeting antibodies [67, 156].

One of the metastasis hypotheses suggests that metastasizing cells move to the peripheral niches occupied by CSCs [196]. According to this hypothesis, TAMs form cell hybrids with tumor cells and travel to distant sites to initiate metastases [197]. The theory of forming a hybrid cell was proposed in 2006 by John Pawelek. He explained this phenomenon as a fusion between a myeloid cell and a tumor cell, leading to a genomic hybridization [198]. This theory was further expanded to include fusion between macrophages and tumor cells in general and TAMs in particular.

It is pertinent to mention that the hybrid cells have a reduced proliferative ability as compared to the parental cell lines. According to the CSCs hypothesis, CSCs form spheroids that migrate out of the primary tumor site *via* the bloodstream or lymphatic circulation and undergo metastases in the niches with appropriate conditions. Macrophages associated with the repair of the injured lesions may serve as niche-forming cells attracting CSCs. Metastatic foci can be further supported by the mutual interaction of these two cell types and the acquisition of TAM characteristics by M2 macrophages to allow tumor growth [199].

The presentation of an antigen is a standard process by which the immune cells eliminate the abnormal cells. However, the hybrid cells have a reduced proliferative potential compared to their parental cell lines. According to the CSC-hypothesis, CSCs form spheroids that migrate out of the primary tumor site via the bloodstream or lymphatic circulation and form metastases in niches under conducive conditions. Macrophages associated with the repair of the injured lesions may serve as niche-forming cells attracting CSCs. Metastatic foci can be further supported by the mutual interaction of these two cell types and the acquisition of TAM characteristics by M2 macrophages to allow tumor growth [200–203].

As antigen presentation is an essential part of the immune response against tumor cells, immune cells such as CD4+ T helper cells and CD8+ cytotoxic T lymphocytes represent one of the most considered and studied processes to eliminate cancer cells. Experimental studies have been done on CSCs and their interactions with lymphocytes in the tumor. For example, in 2019, Masciale et al. described two interesting correlations between CSCs and tumor-infiltrating lymphocytes in NSCLC; between CD3+ T cells and CSCs and between CD8+ T cells and CSCs. These findings are useful for defining the antitumor effects of the cytotoxic CD8+ T cells, and the regulatory CD8+ T cells since CD8+ and CD3+ T cells support the host defense against cancer [5]. In several tumors, cytotoxic T lymphocytes are important predictors of outcomes, both for the disease progression and immunotherapy response [203–205].

CSCs from head and neck squamous cell carcinoma that were positive for CD44 have reduced MHC-I expression compared with the CD44-negative epithelial cancer cells [206]. Similarly, the expression of MHC is lower in CSCs in other cancers [207–210]. On the other hand, no differences in MHC-I expression between CSCs and epithelial cancer cells have been reported [211]. However, the downregulation APCs impede the targeting of CSCs by T cells. This aspect is an essential feature of CSCs in the TME [212]. The ability to regulate APC expression reduces the cell differentiation process and is generally caused by an epigenetic process, such as suppressing histones or DNA methylation. Using a drug-induced DNA demethylation protocol, upregulation of antigen-presenting cells in glioblastoma CSCs has been reported, although the expression remained less than in epithelial cancer cells. This is likely due to the distinct molecular mechanisms that cause different gene expression patterns [208]. However, despite the reduced expression of MHC-I, CSCs remain targets of the immune system, particularly NK cells.

In summary, equilibrium of various signals defines the destiny of CSCs and cancer epithelial cells, and their ability to eradicate or evade an immune response. APCs alone are generally not enough to stimulate T cells. Hence, in the absence of additional signals; T cells destroy themselves or remain inactive. This stimulation is derived from molecules on APCs, i.e., CD80 or CD86 that can bind with T cells. On the other hand, a mechanism of inhibition is PDL1 (B7-H1) presented on tumor cells or APCs. PDL1 binds PD1, inducing the apoptosis or inactivation of T cells. Overexpression of PDL1 is commonly used by cancer cells to inhibit T cells [213]. This aspect has been observed in many cancer types, including glioblastoma and head & neck squamous cell carcinoma [208, 214, 215], thus suggesting an interaction between CSCs and immune cells to have a meaningful impact on promoting or inhibiting T cells. In particular, high levels of PDL1 on CSCs may be interesting to investigate as possible CSC immunotargets [216].

## FUTURE PERSPECTIVES IN CANCER REGARDING TAMs AND CSCs

Surgery is the gold-standard treatment only for cancer in early stages, whereas treatment of advanced cancer stages requires chemotherapy and radiotherapy alone or in combination [217, 218]. However, in many cases, all treatments (chemo-, radio-, and immunotherapy), fail to prevent cancer recurrence [219]. The primary cause of failure in cancer treatment is the emergence of drug resistance that promotes the tumor spreading [220]. Several clinical trials investigate the best cell target to fight cancer, including, but not limited to, CSCs, since CSCs are sustained by other cells, including TAMs (Table 1). Indeed, anti-macrophage drugs such as trabectedin [14, 221, 222], RG7155 (anti-CSF-1R) [223], and an anti-MIF (macrophage migration inhibitory factor) antibody have been developed. However, CSCs are still considered as the most important subpopulation of cells as a novel target due to their integral participation in cancer relapse [224–226]. A recent study has demonstrated that CSCs release cytokines in pro-tumor microenvironment through the generation of CD163+ macrophages like myeloid cells [227]. Moreover, targeting TAMs together with CSCs offer another possible option in treating pancreatic ductal adenocarcinoma to better control cancer progression and avoid tumor dissemination [228]. An important finding is the inhibition of phagocytosis by macrophages through the interaction between signal regulatory protein alpha (SIRPα) and CD47, specific for epithelial cancer cells [229, 230]. Weissman et al. used a monoclonal antibody to block *in vitro* the activity of CD47 for increasing the phagocytosis of the tumor cells leading to a reduction of the tumor growth *in vivo* [228, 229, 231–233].

**Table 1:** Clinical trials targeting CSCs

Drug name	Mechanism	Condition or disease	NCT Number	Current Status
Vismodegib (GDC-0449)	Hedgehog Pathway Inhibitor	Ovarian Cancer	NCT00959647	Completed
	Hedgehog Pathway Inhibitor	Basal Cell Carcinoma	NCT00959647	Completed
	Hedgehog Pathway Inhibitor	Metastatic Colorectal Cancer	NCT00959647	Completed
Sonidegib (LDE225)	Hedgehog Pathway Inhibitor	Medulloblastoma	NCT01708174	Completed
BMS-833923	Hedgehog Pathway Inhibitor	Leukemia	NCT02100371	Completed
MK-0752	Notch pathway inhibitors	Metastatic Breast Cancer	NCT00645333	Completed
RO4929097	Notch pathway inhibitors	Adenocarcinoma of the Pancreas	NCT01122901	Terminated
	Notch pathway inhibitors	Recurrent Adult Brain Tumor	NCT01122901	Terminated
Nirogacestat (PF-03084014)	Notch pathway inhibitors	Desmoid tumors/aggressive fibromatosis	NCT01981551	Active, not recruiting
Crenigacestat (LY3039478)	Notch signaling pathway	Neoplasms	NCT01695005	Completed
	Notch signaling pathway	Lymphoma	NCT01695005	Completed
Demcizumab (OMP-21M18)	Notch pathway inhibitors	Non-Small Cell Lung Cancer	NCT01189968	Completed
Ipafricept (OMP-54F28)	WNT pathway inhibitors	Stage IV Pancreatic Cancer	NCT02092363	Completed
	WNT pathway inhibitors	Pancreatic Cancer	NCT02050178	Completed
Vantictumab (OMP-18R5)	WNT pathway inhibitors	Metastatic breast cancer	NCT01973309	Completed
PRI-724	Wnt signaling pathway blocking	Advanced Solid Tumors	NCT01302405	Terminated
AVID 200	TGF-β inihibitors	Malignant solid tumor	NCT03834662	Active, not recruiting
Fresolimumab (GC1008)	TGF-β inihibitors	Metastatic breast cancer	NCT01401062	Completed
	TGF-β inihibitors	Stage IA Non-Small Cell Lung Carcinoma	NCT02581787	Recruiting
NIS793	TGF-β inihibitors	MPN (Myeloproliferative Neoplasms)	NCT02947165	Active, not recruiting
	TGF-β inihibitors	Lung cancer	NCT02947165	Active, not recruiting
	TGF-β inihibitors	Hepatocellular Cancer	NCT02947165	Active, not recruiting
	TGF-β inihibitors	Colorectal Cancer	NCT02947165	Active, not recruiting
	TGF-β inihibitors	Pancreatic Cancer	NCT02947165	Active, not recruiting
Ruxolitinib	JAK inihibitors	Metastatic breast cancer	NCT01348490	Completed
	JAK inihibitors	Myeloproliferative neoplasms	NCT01348490	Completed
AZD4205	JAK inihibitors	Advanced non-small cell lung cancer	NCT03450330	Completed
SAR245409	PI3K and mTOR inihibitors	Advanced or metastatic solid tumors	NCT01240460	Completed
Matuzumab (EMD 72000)	EGFR inhibitors	Non small cell lung carcinoma	NCT00753246	Completed

Clinical trials evaluating drugs for the selective inhibition of cancer stem cells in different solid tumors and hematologic malignancies.

Cioffi et al. have extended this concept in a pancreatic cancer model, describing a novel therapy that induced phagocytosis of CSCs [230]. This approach has been tested in pancreatic cancer with encouraging results and hence necessitates further studies must be performed in other solid tumors.

Another approach recently taken into consideration as a prospective approach in cancer therapies is nanomedicine. The term “nanos” refers to preparations with non-sized particles ranging in size between 1–100 nm in diameter [229–235]. Their main composition of such preparations may include lipids, proteins, or polymer [236, 237], and they may be used as a *loader* of drugs or genes or for diagnostic purposes [237, 238]. *Ex vivo* loading of patients’ cells, i.e., macrophages, monocytes, or MSCs, with a loaded nanodrug, are considered to be the most effective approach for nanodrug delivery without compromising the cell viability and mobility [239]. These drug-loaded cells can home-in to the tumor site or areas of inflammation [240] focusing on immunocytes and stem cells due to their intrinsic neoangiogenic capacity around inflammatory or tumor sites.

The cell-based targeted nanoparticles have been described in several studies and can adhere to antibodies, peptides, etc. [235, 239, 241, 242–255]. Their targets are generally membrane-bound and intracellular receptors [256, 257], or mitochondria [258]. The most complicated aspect of this interaction is the space between the target and the molecules that needs to be bound: This space must be no larger than a few nm [259]; otherwise, the interaction would not be effective. This aspect is easier to address *in vitro*. It may represent a problem *in vivo*, as the characteristics of the TME are more varied and are different from the media commonly used *in vitro*. It has been shown that the target of nanoparticles does not produce an increment of the particles able to bind cancer cells *in vivo* [260]. This aspect suggests that the target of cell-based nanoparticles in oncology has been over-considered, while the physical characteristics of nanoparticles must receive more focus [261]. In addition to these aspects, the microenvironment, particularly the cell-to-cell interactions and the cell-immune system interactions, should also be taken into consideration, as they may constitute a huge obstacle for the optimization of this approach against cancer. The synthesis of oral nanodrugs will likely be more useful than intravenous solutions, and this may represent the main factor in the clinical use of nanodrugs to treat cancer [262, 263]. However, several barriers are required to be overcome before interaction between nanoparticles and the components of the TME.

Interestingly, the intrinsic phagocytic activity of macrophages has been exploited to load anti-cancer nanodrugs. One of the first accepted therapies in this field involved the innovative delivery of bioactive proteins into macrophages to treat neurodegenerative diseases [264, 265]. This therapeutic approach is based on nanozymes, which can be rapidly internalized by monocyte-derived macrophages and released in an active form within 24 hours. In a mouse model of Parkinson’s disease, the injected monocyte-derived macrophages were able to home-in to the brain, and there was an overall reduction in oxidative stress. Macrophages loaded with a DNA plasmid encoding for catalase had a similar effect as exosomes secreted by the macrophages and attenuated oxidative stress in neurons [266]. This strategy exhibits promise in improving motor neuron functions in mouse models of Parkinson’s disease. These nano-carriers are particularly useful due to their ability to cross the blood–brain barrier (BBB) [267]. The same approach has been used for antiretroviral drugs against HIV, encapsulating them in biodegradable nanoparticles loaded *ex vivo* into monocyte-derived macrophages. These macrophages were able to deliver drugs across the BBB and inhibit HIV infection in the brain. More recently, given these promising effects, macrophage-loading with drugs has been extended to antiviral drugs such as ritonavir, indinavir, and efavirenz [268].

A novel approach to improving nanodrug capture efficiency by macrophages for anti-HIV therapy is being developed [269]. This approach uses a well-known chemoattractant for macrophages, N-formyl-methionyl-leucyl-phenylalanine peptide conjugated onto PEGylated nanoparticles. This approach was useful for nanodrug internalization by peritoneal macrophages, which are the primary HIV reservoirs. At molecular level, macrophage scavenger receptors are actively involved in capturing circulating nanodrugs [270]. These studies demonstrated the importance of using nanoparticles that can be captured by macrophage scavenger receptors.

## CONCLUSIONS

In-depth understanding of interaction between TAMs and CSCs is needed to develop novel treatment strategies in future. In this direction, researchers have already reported the presence of CSCs in many solid tumors as the leading cause of cancer relapse and chemotherapeutic drug resistance. In addition to this subpopulation of cells, macrophages and other immune cells also participate in interactions that may aid or impede the fight against cancer. For this reason, the targeting TAMs offer a novel treatment option against cancer. The different therapeutic approaches developed to target TAMs include the depletion, blockade of monocyte/macrophage recruitment, reprogramming of TAMs into pro-inflammatory M1-like macrophages, and neutralizing the products of TAMs [271]. Although most TAM-targeting strategies are in the pre-clinical stages, several factors used for TAMs depletion have already been tested in clinical trials [271, 272]. However, the effects of these novel treatments targeting TAMs on checkpoint blockade-based immunotherapies must be further investigated [273]. We believe that targeting TAMs may trigger various stromal reactions in the tumor milieu that are difficult to predict, even if the variability from patient to patient is kept as a consideration. Targeting TAMs could not only inhibit the TME, but also renovate the tumor “soil” to build a tumor-suppressive microenvironment, thereby suppressing tumor development. This strategy may become an effective therapeutic intervention that may be used either alone or in combination with other therapeutic strategies to treat cancer [273].

In summary, generating new information about the interaction between TAMs and CSCs will be one of the most important challenges for the development of more effective targeted cancer therapies.

## Abbreviations

TAM: tumor-associated macrophages
ALDH: aldehyde dehydrogenase
ARGI: arginase I
BBB: blood–brain barrier
CSCs: Cancer stem cells
COX-2: cyclooxygenase 2
CTLA-4: cytotoxic T lymphocyte-associated protein 4
EGF: epidermal growth factor
EMT: epithelial-to-mesenchymal transition
GSLCs: glioma stem-like cells
HCC: hepatocellular carcinoma
NOS: nitric oxide synthase
PDGF: platelet-derived growth factor
PD-1: receptors programmed cell death
TME: tumor microenvironment
Tregs: recruit regulatory T cells
